# Exploration of Social Benefits for Tourism Performing Arts Industrialization in Culture–Tourism Integration Based on Deep Learning and Artificial Intelligence Technology

**DOI:** 10.3389/fpsyg.2021.592925

**Published:** 2021-02-16

**Authors:** Ruizhi Zhang

**Affiliations:** School of Arts, Hunan City University, Yiyang, China

**Keywords:** social benefit, BP neural network, towns with cultural and tourism characteristics, fuzzy comprehensive analysis method, cultural inheritance and protection

## Abstract

As a product of the tourism performing arts industry in culture–tourism integration development, to develop a featured culture–tourism town is a new trend for tourism development in the new era. To analyze the social benefit of the culture–tourism industry, in this study, an artificial intelligence model for social benefit evaluation is constructed based on backpropagation (BP) neural network and fuzzy comprehensive analysis, with Yiyang Town taken as an example. The criterion layer in the model includes three indexes (life benefit G1, environmental benefit G2, and economic benefit G3), and the index layer contains 11 indexes (H1–H11). The weight values of cultural inheritance and protection, ecological environment improvement, and commercial economy development to the social benefit of the town are 0.522, 0.570, and 0.424, respectively. For G1, 41.20% is excellent; for G2, 39.5% is excellent; and for G3, 40.5% is good. In general, 30.76% of the total social benefit is excellent, with 37.69% being good, 21.48% being qualified, and 10.07% being unqualified. It is inferred that the total social benefit level of Yiyang Town is good according to the constructed model. Therefore, the culture inheritance and protection, the ecological environment improvement, and the commercial economy development are the key evaluation factors of social benefit.

## Introduction

As time goes by, Chinese tourists can no longer be attracted by purely scenic spots. Industry integration, as a more advanced mode for the vigorous development of the industry, has become an inevitable trend (Kim et al., 2019; Urien-Lefranc, 2020; Yang and Wang, 2020). Tourism performing arts is one of the typical forms in culture–tourism integration development in recent years. Being an important carrier of cultural heritage, it carries forward Chinese culture during tourism (Samora-Arvela et al., 2020). In recent years, the tourism performing arts is increasingly energetic. According to relevant data from 2013 to 2017, the domestic number of channels for the tourism performing arts program increased from 187 to 268, up by 43%. The number of tourism performing arts programs increased from 53,336 to 85,753, with an increase of 61%. Audience of tourism performing arts increased from 27.89 million to 68.21 million, up by 145%. The box-office revenue of tourism performing arts increased from 2.26 billion yuan to 5.15 billion yuan, with an increase of 128% (Ranasinghe and Cheng, 2018). Especially in the past 2 years, with the market being more mature, tourism performing arts projects have seen faster development. The culture–tourism town is a new tourism product with the integration of cultural and performing industries (Zhang et al., 2020). As one of the culture–tourism towns in Hunan Province, the transportation infrastructure in Yiyang has experienced vigorous progression in recent years, which further highlights its regional advantages. In addition, many culture–tourism projects of Yiyang characteristics have been constructed, such as Tianyi Muguo (a resort characterized by the things made of wood), tea-horse ancient road, tea-scented flower sea (a place characterized by all kinds of flowers), and Anhua Meishan Cultural Ecological Park, which makes Yiyang more popular in tourists (Wu and Wu, 2017; Drius et al., 2019). Therefore, the Yiyang Town in Yiyang region is taken as an example in the study.

Fuzzy comprehensive evaluation based on fuzzy mathematics is a comprehensive evaluation method which adopts fuzzy relation synthesis to quantify some factors with unclear boundaries (Wu and Wu, 2017; Chen, 2019; Yuan and Wu, 2020). This method is mainly applicable to evaluation objectives with multiple variables and fuzziness. Through the construction of a reasonable evaluation system, the evaluation factor is assigned a certain value according to the score given by the expert, and then reliable results are obtained through analysis (Su et al., 2016; Obschonka et al., 2018). Traditional artificial intelligence mainly contains pattern analysis, machine learning, and data mining. As a hot topic in the field of artificial intelligence, deep learning has made breakthroughs in such fields as large-scale speech recognition and large-scale image retrieval (Li and Cao, 2018). The BP neural network is one of the most effective multilayer deep learning methods among them, which has complex pattern classification capability and excellent multidimensional function mapping capability, and can solve different problems that simple perceptron cannot solve. Further, it can also continuously adjust the network weight value to make the final output of the network as close as possible to the expected output (Rogoza et al., 2018; Li et al., 2019). From the perspective of inbound tourism demand, Shi (2020) used deep learning BP neural network to extract 7 influence factors to construct the eigenvector and predicted the number of inbound tourists in Yangjiang in 2018–2019. The results showed that the mean square error and R2 coefficient were 0.011695 and 0.94744, respectively, which were acceptable. Based on the deep learning BP neural network, Li (2020) constructed an artificial intelligence prediction model for the temporal and spatial distribution of tourists and collected the spatial and temporal distribution data from five aspects: the probability distribution of tourists' destinations, the transfer probability between tourist attractions, the scenic spot time distribution, the moving time between scenic spots, and the scenic spot area. It is found that this artificial intelligence model had high prediction accuracy and is suggested in the temporal and spatial distribution prediction of tourists. Therefore, in the study, deep learning BP is combined with the fuzzy comprehensive evaluation method to design an artificial intelligence model for social benefit evaluation.

## Materials and Methods

### The Selection of the Research Subject

**Figure 1:**
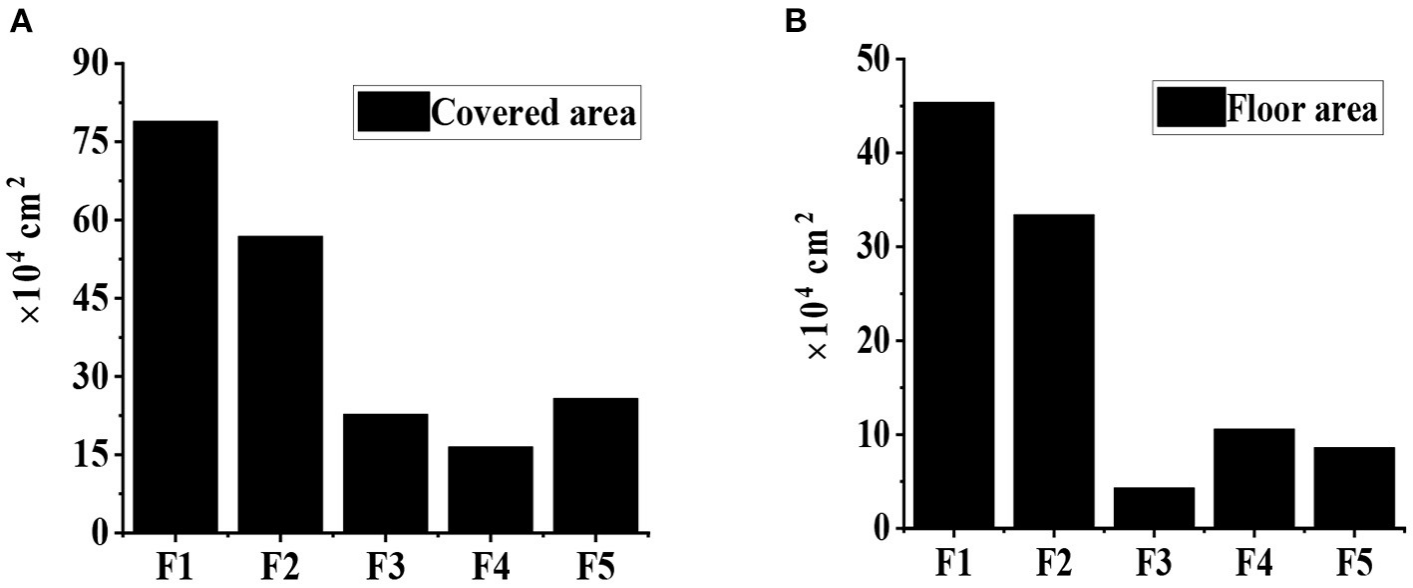
The floor space and building area of function zones in Yiyang culture–tourism town. **(A)** The floor space; **(B)** is the building area.

In this study, the Yiyang culture–tourism town in Yiyang region is selected as the research subject. The function zones of this town include health and health community (F1), agricultural sightseeing and leisure zone (F2), outdoor ecological experience zone (F3), waterfront leisure zone (F4), and folk performing arts culture experience zone (F5). As shown in Figure 1, the healthcare community covers a floor space of 789,167m^2^ and a building area of 453,717m^2^, respectively; the agricultural sightseeing and leisure zone covers a floor space of 568,807m^2^ and a building area of 334,107m^2^, respectively; the outdoor ecological experience zone covers a floor space of 227,362 square meters and a building area of 43,189m^2^, respectively; the waterfront leisure area covers a floor space of 164,911m^2^ and a building area of 105,726m^2^, respectively; and the folk performing arts cultural experience covers a floor space and a building area of 257,737 and 86,081m^2^, respectively.

### Definition of Relevant Concepts

#### The Culture-Tourism Town

**Figure 2:**
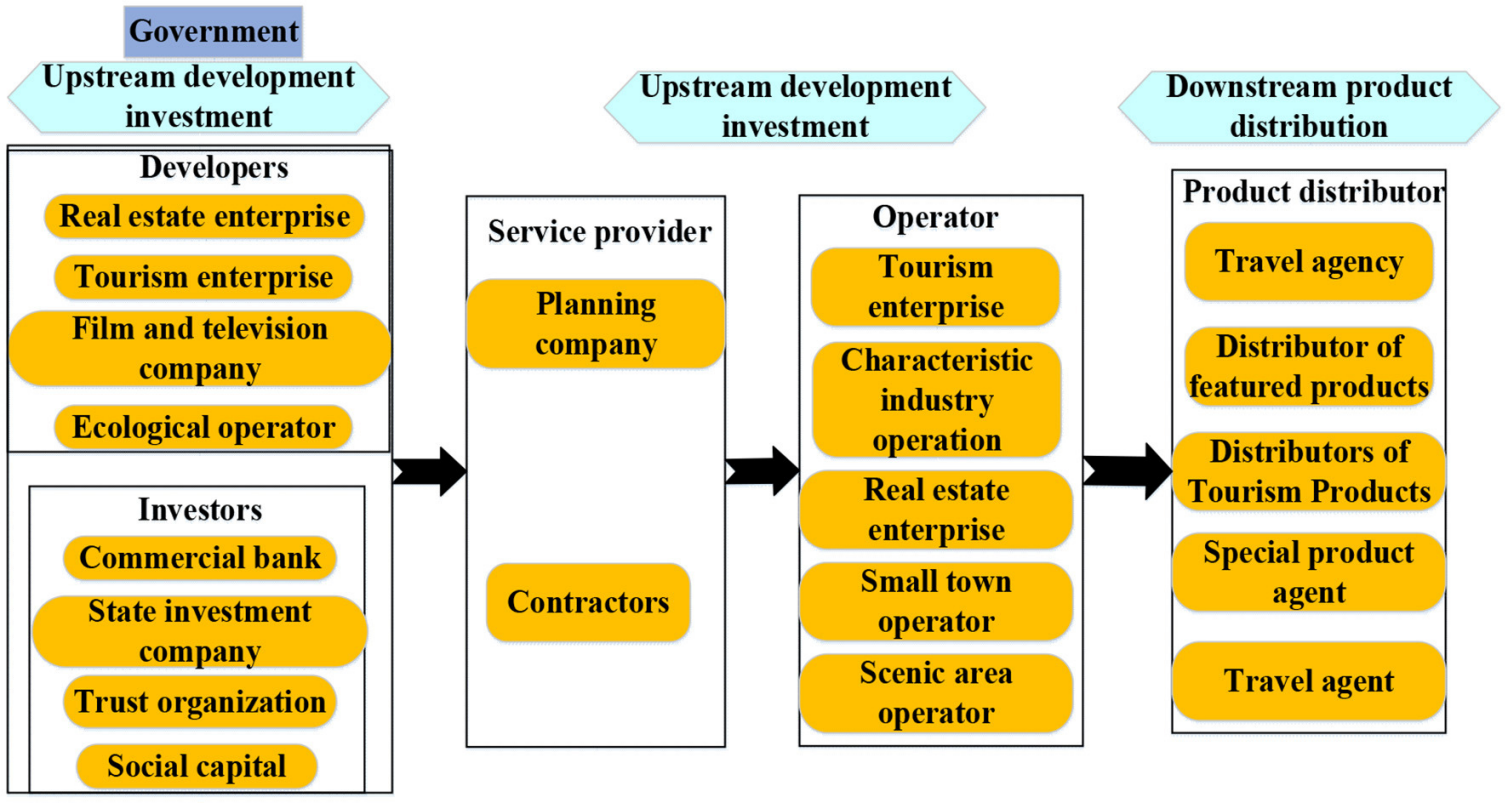
The industry chain of the culture–tourism town.

A culture-tourism town refers to a town with clear industrial orientation, cultural characteristics, tourism characteristics, and certain community functions, which is different from an administrative town. It can turn the idle resources of rural areas, such as green mountains and clear waters, pastoral lifestyle, and traditional culture, into economic advantages. Besides, it can also drive the flow of urban capital to the countryside, thus improving rural infrastructure, public services, and environmental health. Furthermore, it also creates new jobs for farmers (Yi et al., 2019). In general, the culture–tourism town is a town involving culture inheritance in tourism development, which combines various elements such as food, clothing, housing, and transportation. Nowadays, there has been a preliminary industrial chain for culture–tourism town development, as shown in Figure 2, including three stages which are upstream development and investment, midstream planning and operation, and downstream product distribution. The upstream development investment includes investors and developers, the midstream planning and operation includes service providers and operators, and the downstream product distribution includes product distributors and distribution channels, and at the end are the users.

#### Social Benefit of Small Towns With Culture–Tourism Characteristics

Social benefit refers to the maximum use of limited resources, which aims to satisfy growing material and cultural needs of people in society. To be specific, it is the total contributions of a certain project to employment, living standard, and social welfare. The construction of the culture–tourism town improves local employment, living standard, and regulation system (Kim et al., 2020). What is more, the coordination and cooperation between government supervision and market economy is realized during the process.

### Evaluation Indexes of Social Benefit of the Culture–Tourism Town

**Figure 3:**
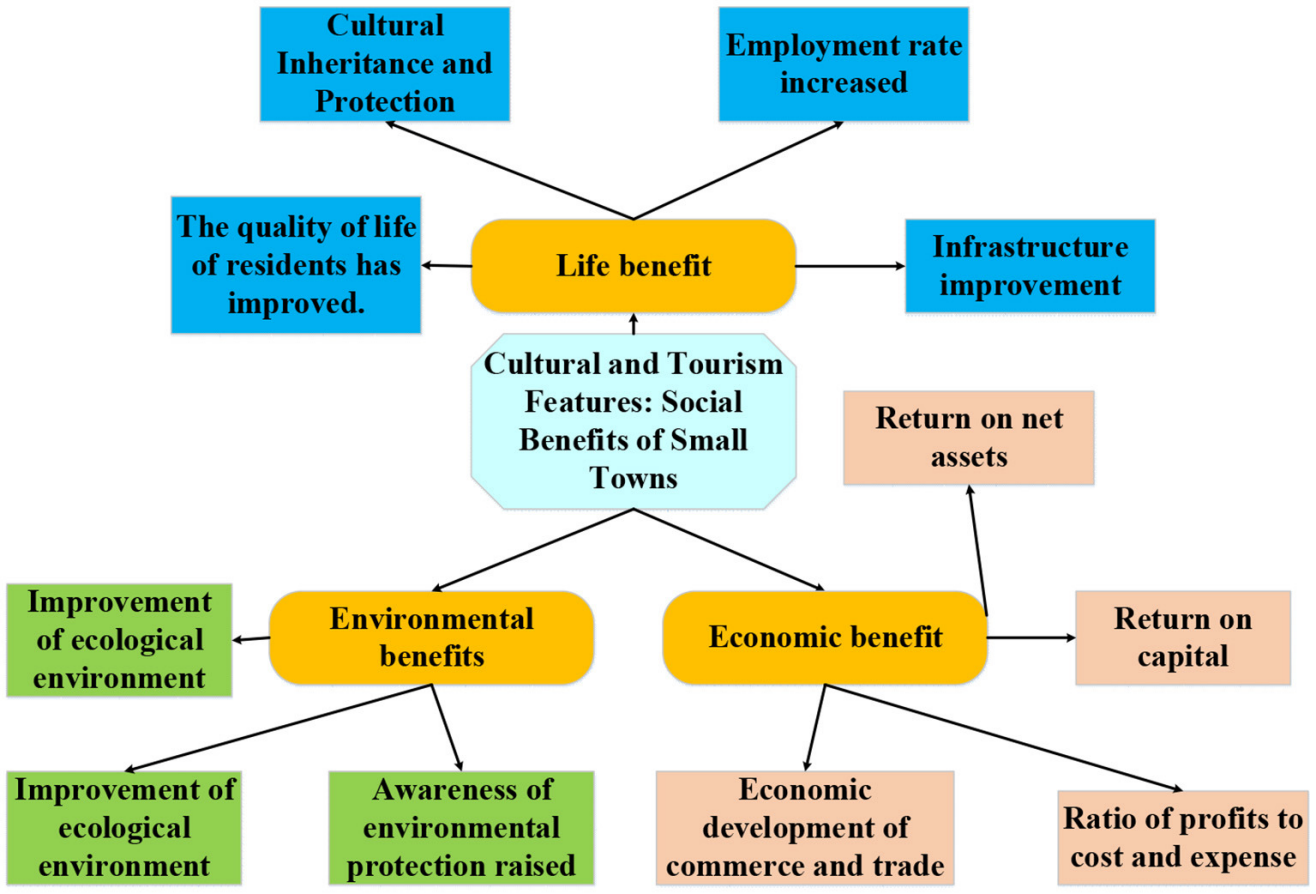
The evaluation indexes of social benefit of the culture–tourism town.

At present, although there are an increasing number of scholars analyzing the benefit of the culture–tourism town, with some relevant index systems established, there are few studies on the social benefit of the culture–tourism town. Most of the literature focuses on the economic benefit (Ramsey and Malcolm, 2018; Yousif, 2018). Therefore, with China's national conditions and policy development taken into account, the social benefit evaluation indexes are selected by referring to previous literature. The results are shown in Table 1. According to these indexes, the social benefit evaluation index system of the culture–tourism town is constructed, as shown in Figure 3.

**Table 1:** Evaluation index of social benefit of small towns with culture–tourism characteristics.

	**Number**	**Social benefit evaluation index**	**Definition**
G1	H1	Cultural inheritance and protection	Whether the construction can coordinate the relationship between local cultural resources and town development
	H2	Employment rate increase	Whether a large number of jobs are created for local people
	H3	The life quality improvement	Whether the local economy is growing faster, residents' living quality is improving, and the income is rising
	H4	Infrastructure improvement	Whether infrastructure such as transportation, water supply, heating, and wireless networks has been improved
Environment benefit	H5	Ecological environment improvement	Whether local water, air, noise pollution, and other environmental problems have been properly dealt with
	H6	Green vegetation protection	Whether the economic value of the local green vegetation is exploited and well-preserved
	H7	Raised environmental protection awareness	Whether local residents feel the benefit brought by the beautiful environment and can be more active in protecting the local ecological environment
G3	H8	Commercial economy development	Whether it can attract a large number of foreign tourists to promote the local and surrounding accommodation, catering, and other related industries
	H9	Return on equity (R0E)	R0E = project net profit/net assets ×100%, which can reflect the profitability of the project
	H10	Ratio of profits to cost and expense (RPCE)	RPCE = total profit/total cost ×100%, which can reflect the profit brought by operating costs
	H11	Return on capital (ROC)	ROC = net profit/average capital ×100%, which can reflect the ability to use capital to bring income

### Artificial Intelligence Evaluation Model Based on BP-Fuzzy Comprehensive Analysis

Many evaluation indexes are selected in this study, all of which are qualitative factors with fuzziness. In addition, there are differences in the influence degree of each index on the social benefit of the culture–tourism town. Therefore, the fuzzy processing on each index is carried out. Then, the fuzzy factors are quantitatively analyzed to determine the expected output value of the deep learning BP neural network (Wu et al., 2020). Finally, the model is constructed through sample training and learning. The specific process is as follows.

First, the fuzzy comprehensive evaluation method is used to deal with the evaluation indexes.

Step 1: Establishment of a set of evaluation indexes. After the evaluation indexes are selected, they are all put into a factor set U, which can be expressed as follows.

(1) $$\begin{array}{r} U=\left\{u_{1},u_{2},u_{3},\cdots \;,u_{n}\right\} \end{array}$$

where *u*_*i*_(*i* = 1, 2, 3, ⋯ , *n*) is the evaluation index factor and *n* represents the number of evaluation factors at the same level.

Step 2: Establishment of a comment set. According to the evaluation results of the evaluation system, the evaluation set V can be expressed as follows.

(2) $$\begin{array}{r} V=\left\{v_{1},v_{2},v_{3},\cdots \;,v_{m}\right\} \end{array}$$

where *v*_*j*_(*j* = 1, 2, 3, ⋯ , *m*) represents the evaluation result of the jth type and m represents the number of evaluation grades.

Step 3: Establishment of the weight coefficient of the evaluation index. The analytic hierarchy process is used to classify the evaluation indexes. The first layer is the evaluation target layer, and the second layer is the criterion layer, which can be expressed as follows.

(3) $$\begin{array}{r} U=\left\{u_{1},u_{2},u_{3},\cdots \;,v_{k}\right\} \end{array}$$

The third layer is the quantification of evaluation indexes.

(4) $$\begin{array}{r} U_{i}=\left\{u_{1},u_{2},u_{3},\cdots \;,v_{k}\right\},\left(i=1,2,3\cdots \;,n\right) \end{array}$$

Then, the index weight of each layer is obtained by building a judgment matrix and normalization. In this study, the weight of the second layer is set as G, which can be expressed as follows.

(5) $$\begin{array}{r} G=\left\{g_{1},g_{2},g_{3},\cdots \;,g_{k}\right\} \end{array}$$

The weight of the index of the third layer is set as follows.

(6) $$\begin{array}{r} G_{i}=\left\{g_{i1},g_{i2},g_{i3},\cdots \;,g_{in}\right\} \end{array}$$

where *G*_*i*_ ≥ 0, *G*_*ij*_ ≤ 1, and *G*_*i*_ + *G*_*ij*_ = 1.

Step 4: First-level fuzzy comprehensive evaluation. In this study, the single-level evaluation method (Wang, 2020) is used to obtain the membership matrix of each evaluation index, which can be as follows.

(7) $$\begin{array}{r} R_{i}=\left[\begin{array}{cccc} r_{i11} & r_{i12} & \cdots & r_{i1q}\\ r_{i21} & r_{i22} & \cdots & r_{i2q}\\ r_{i31} & r_{i32} & \cdots & r_{i3q}\\ r_{in1} & r_{in2} & \cdots & r_{inq} \end{array}\right] \end{array}$$

where *i* = 1, 2, ⋯ , *k*, q represents the number of evaluation criteria, and n represents the number of evaluation factors. Through the combination of the weight coefficient of each evaluation index with the membership degree matrix, the evaluation result vector of the index factor can be expressed as follows.

(8) $$\begin{array}{r} B_{i}=gi\times R_{i}=\left\{g_{i1},g_{i2},g_{i3},\cdots \;,g_{in}\right\}\times \left[\begin{array}{cccc} r_{i11} & r_{i12} & \cdots & r_{i1q}\\ r_{i21} & r_{i22} & \cdots & r_{i2q}\\ r_{i31} & r_{i32} & \cdots & r_{i3q}\\ r_{in1} & r_{in2} & \cdots & r_{inq} \end{array}\right]\\ =\left(b_{1},b_{2},b_{3},\cdots \;,b_{iq}\right) \end{array}$$

Step 5: Second-level fuzzy comprehensive evaluation. Based on the first-level fuzzy comprehensive evaluation results, there is also a corresponding relationship between each evaluation index, so the evaluation results of each index are integrated using fuzzy mathematics calculation. Then, the final evaluation result vector obtained is as follows.

(9) $$\begin{array}{r} B=G\times {\left\{B_{1},B_{2},B_{3},\cdots \;,B_{K}\right\}}^{T}=\left(g_{1},g_{2},g_{3},\cdots \;,g_{k}\right)\\ \times {\left(B_{1},B_{2},B_{3},\cdots \;,B_{K}\right)}^{T} \end{array}$$

**Figure 4:**
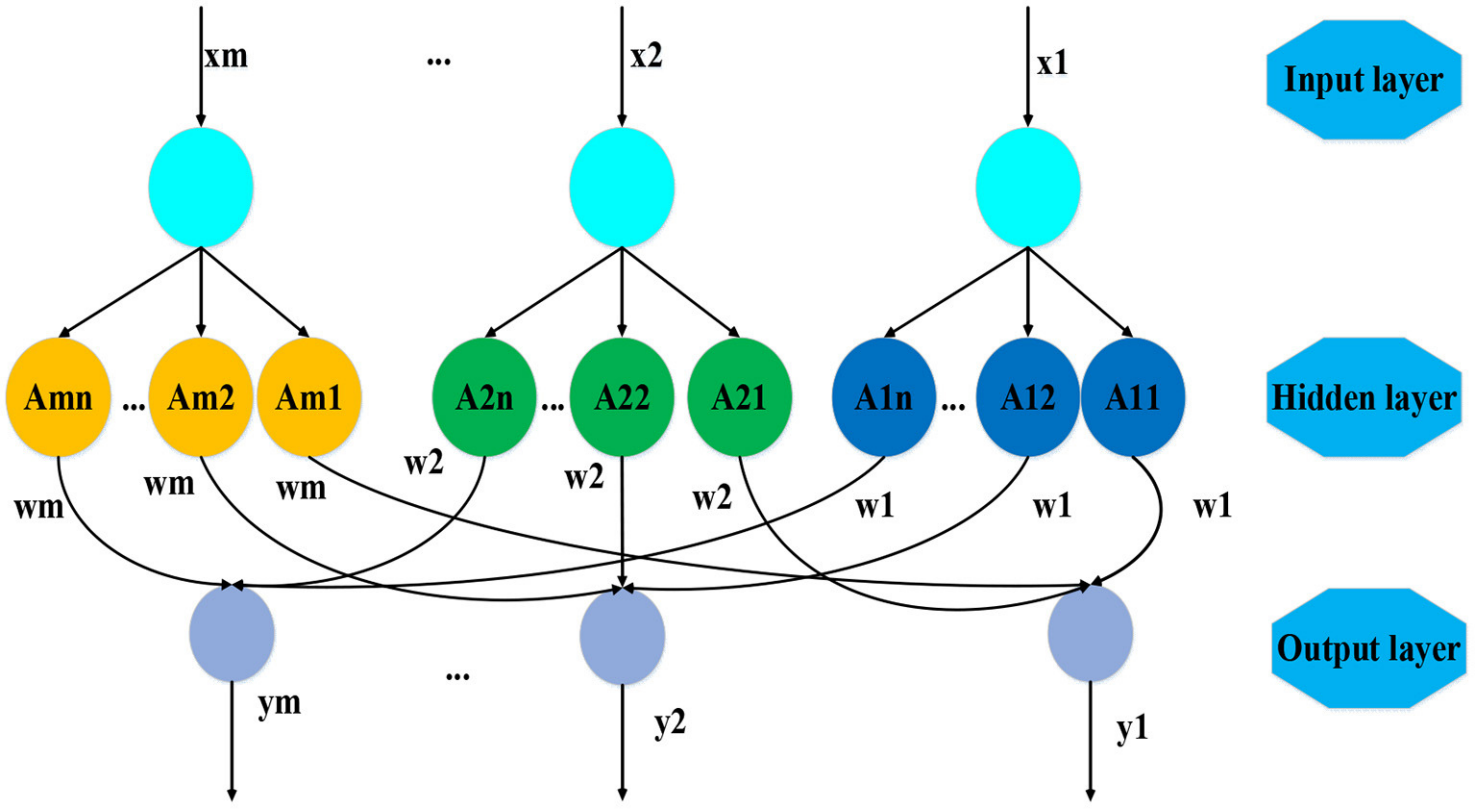
The three-layer BP neural network structure.

According to the above steps, the weight value of each index has a great impact on the final evaluation result. Therefore, the BP neural network is used in this study to determine the weight value of the comprehensive evaluation index. First, the structure of the neural network used to determine the weight is constructed, as shown in Figure 4, which is divided into the input layer, the hidden layer, and the output layer.

Step 6: The input layer. The input element is the value of each evaluation index. The dimensionality of each index is different, so the values of each index are converted into non-dimensional standard values first. There are m neurons in the input layer, and the input and output of the neurons can be expressed as follows.

(10) $$\begin{array}{r} I\begin{array}{c} 1\\ i \end{array}=x_{i} \end{array}$$

(11) $$O_{ij}^{1}=x_{i}$$

where *i* = 1, 2, ⋯ , *m*, *j* = 1, 2, ⋯ , *n*, $I_{i}^{1}$ represents the input, and $O_{ij}^{1}$ is the output.

Step 7: The hidden layer. The hidden layer differentiates the input of the input layer and obtains the membership value of each input level according to the membership function. In the constructed BP network, there are *n* comment levels and a total of *m*×*n* neurons. In this study, trigonometric functions (Zhang et al., 2019) are introduced to express membership functions, which can be as {*A*_*ij*_} = {*NB, NS, N, PS*} = {excellent, good, qualified, unqualified}. Therefore, *n* = 4, then the evaluation index membership function is calculated as follows.

When *j* = 1, the corresponding membership function parameters are *a*_1_ and *a*_2_.

(12) μ1x={1x≤a1a2−xa2−a1a1≤x≤a20x≥a2

where μ_1_*x* represents a membership function. When *j* = *n*, the corresponding membership function parameters are *a*_*n*_ and *a*_*n*−1_.

(13) μnx={0x≤an−1x−anan−an−1an−1≤x≤an1x≥an

When 1 < *j*<*n*, the corresponding membership function parameters are *a*_*j*_, *a*_*j*−1_, and *a*_*j*+1_.

(14) μjx={0x≤aj−1 or x≥aj+1x−aj−1aj−aj−1aj−1≤x≤ajaj+1−xaj+1−ajaj≤x≤aj+1

According to the above calculation method, the output of the hidden layer can be obtained as follows.

(15) $$O_{ij}^{2}=A_{ij}\left(x_{i}\right)$$

where *A*_*ij*_(*x*_*i*_) represents the numerical membership function of the jth comment level and $O_{i}^{2}$ represents the membership value of each level.

Step 8: The output layer. The output layer mainly evaluates each input vector and obtains the corresponding evaluation vector according to the value of the evaluation level. Then, the output can be expressed as follows.

(16) $$\begin{array}{r} O_{i}^{3}=\overset{m}{\underset{j=1}{\sum }}w_{j}I\begin{array}{c} 3\\ ij \end{array} \end{array}$$

where *i* = 1, 2, 3, ⋯ , *m*, *j* = 1, 2, 3, ⋯ , *n*.

**Figure 5:**
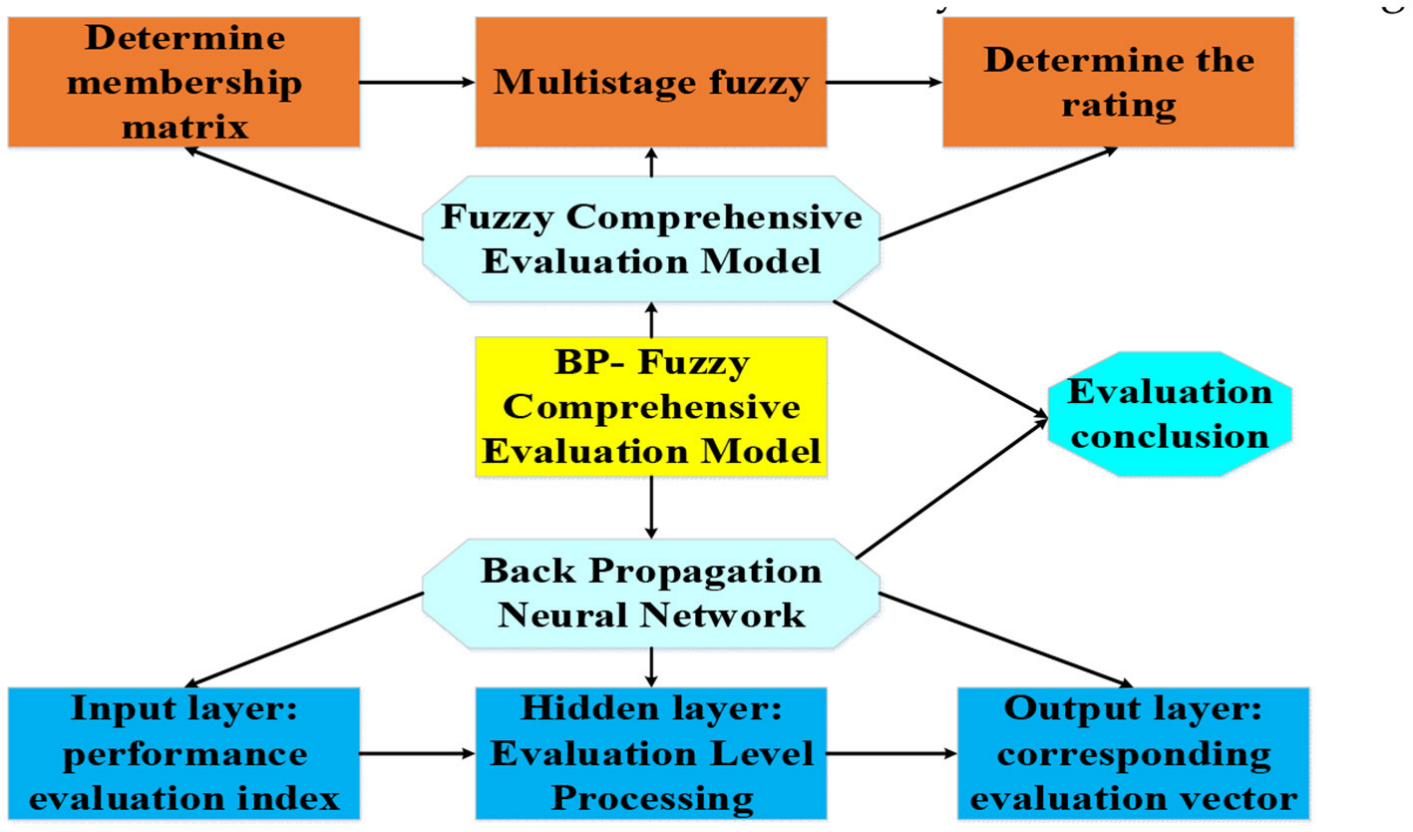
The construction of BP-fuzzy comprehensive model for social benefit evaluation of the culture–tourism town.

To sum up, the structure of the BP-fuzzy comprehensive evaluation model for social benefit of small towns with culture–tourism characteristics in this study are shown in Figure 5.

## Results

### Calculation of Each Index Weight

#### Calculation of the Weight Value of the W–G Judgment Matrix

According to the model constructed in this study, the index system is firstly divided into three layers. The first level is the target layer: social benefit of the culture–tourism town, recorded as W. The second level is the criterion layer: G1, G2, G3. The third level is the index layer which contains indexes of the cultural inheritance and protection H1, the employment rate increase H2, the quality-of-life promotion H3, infrastructure improvement H4, the ecological environment improvement H5, green vegetation protection H6, the environmental awareness improvement H7, the commercial economy development H8, ROE H9, RPCE H10, and ROC H11.

**Figure 6:**
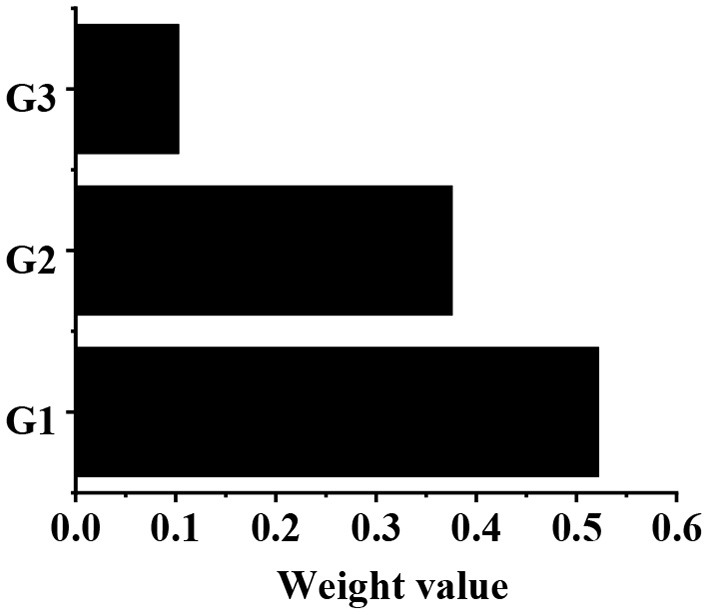
The weight values of G1, G2, and G3 relative to the target layer W.

The calculation of the weight value of the W–G judgment matrix is shown in Table 2. After calculation, g1 = 8.333, g2 = 6, and g3 = 1.643. After normalization, the eigenvector can be obtained as follows: $\vec{g}={\left(0.522,0.376,0.103\right)}^{T}$. According to the consistency test, CI = 0.074, and CR = 0.052, which are both <0.1. The weight values of G1, G2, and G3 relative to the target layer W are 0.522, 0.376, and 0.103, respectively, as shown in Figure 6.

**Table 2:** Calculation of the weight value of the W-G judgment matrix.

**W**	**G1**	**G2**	**G3**
G1	1	1/2	7
G2	2	1	3
G3	1/7	1/3	1

#### The Calculation of Weight Value in the G–H Judgment Matrix

**Figure 7:**
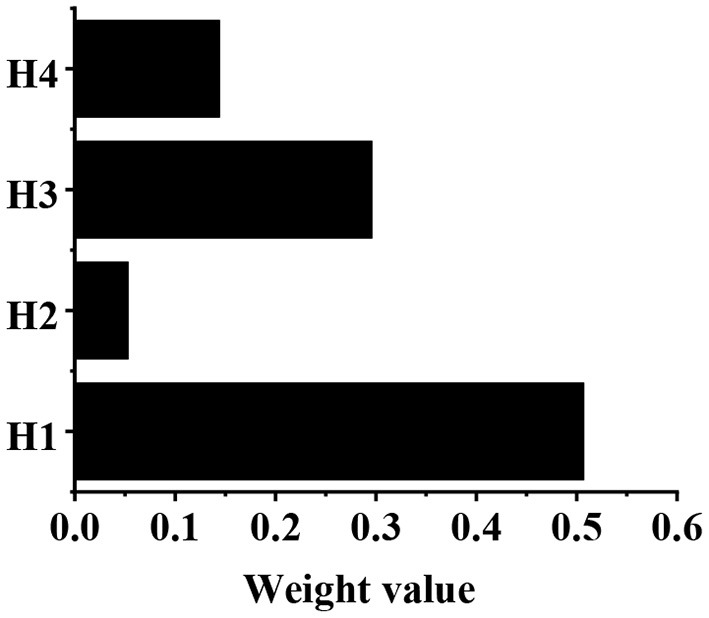
The weight values of H1, H2, H3, and H4 relative to G1.

**Figure 8:**
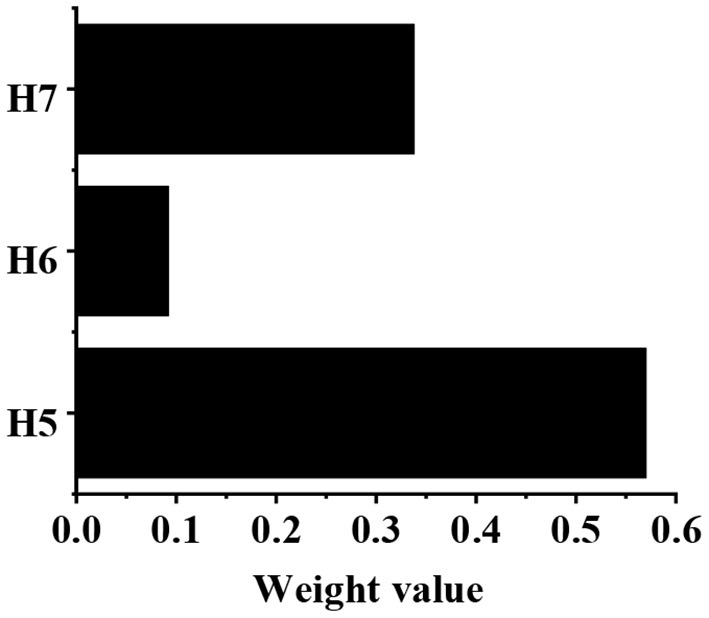
The weight values of H5, H6, and H7 relative to G2.

The calculation of weight value in the G1–H judgment matrix is shown in Table 3. After calculation, g4 = 16, g5 = 1.676, g6 = 9.333, and g7 = 4.533. After normalization, the eigenvector can be obtained as follows: $\vec{g}={\left(0.507,0.053,0.296,0.144\right)}^{T}$. According to the consistency test, CI = 0.050, and CR = 0.039, which are both <0.1. Then, the weight values of H1, H2, H3, and H4 relative to G1 are shown in Figure 7, which are 0.507, 0.053, 0.296, and 0.144, respectively. The calculation of weight value in G2-H judgment matrix is shown in Table 4. After calculation, g8 = 9, g9 = 1.450, g10 = 5.333. After normalization, the eigenvector can be obtained as follows. According to the consistency test, CI = 0.047, and CR = 0.062, which are both less than 0.1. Then, the weight values of H5, H6, and H7 relative to G2 are shown in Figure 8, which are 0.570, 0.092 and 0.338, respectively.

**Table 3:** Calculation of weight value of the G1–H judgment matrix.

**G1**	**H1**	**H2**	**H3**	**H4**
H1	1	7	3	5
H2	1/7	1	1/5	1/3
H3	1/3	5	1	3
H4	1/5	3	1/3	1

**Table 4:** Calculation of weight value of the G2–H judgment matrix.

**G2**	**H5**	**H6**	**H7**
H5	1	5	3
H6	1/5	1	1/4
H7	1/3	4	1

**Figure 9:**
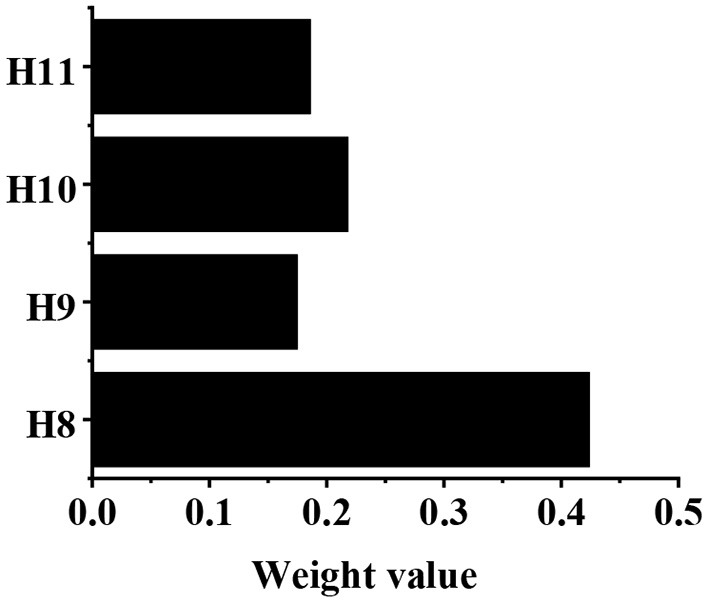
The weight values of H8, H9, H10, and H11 relative to the G3.

The calculation of weight value in the G3–H judgment matrix is shown in Table 5. After calculation, g11 = 11, g12 = 4.45, g13 = 5.666, and g14 = 4.833. After normalization, the eigenvector can be obtained as follows: $\vec{g}={\left(0.424,0.175,0.218,0.186\right)}^{T}$. According to the consistency test, CI = 0.081, and CR = 0.056, which are both <0.1. Then, the weight values of H8, H9, H10, and H11 relative to the G3 are shown in Figure 9, which are 0.424, 0.175, 0.218, and 0.186, respectively.

**Table 5:** Calculation of weight value of the G3–H judgment matrix.

**G3**	**H8**	**H9**	**H10**	**H11**
H8	1	5	3	2
H9	1/5	1	1/4	3
H10	1/3	4	1	1/3
H11	1/2	1/3	3	1

#### The Weight Value of the W–H Index

**Figure 10:**
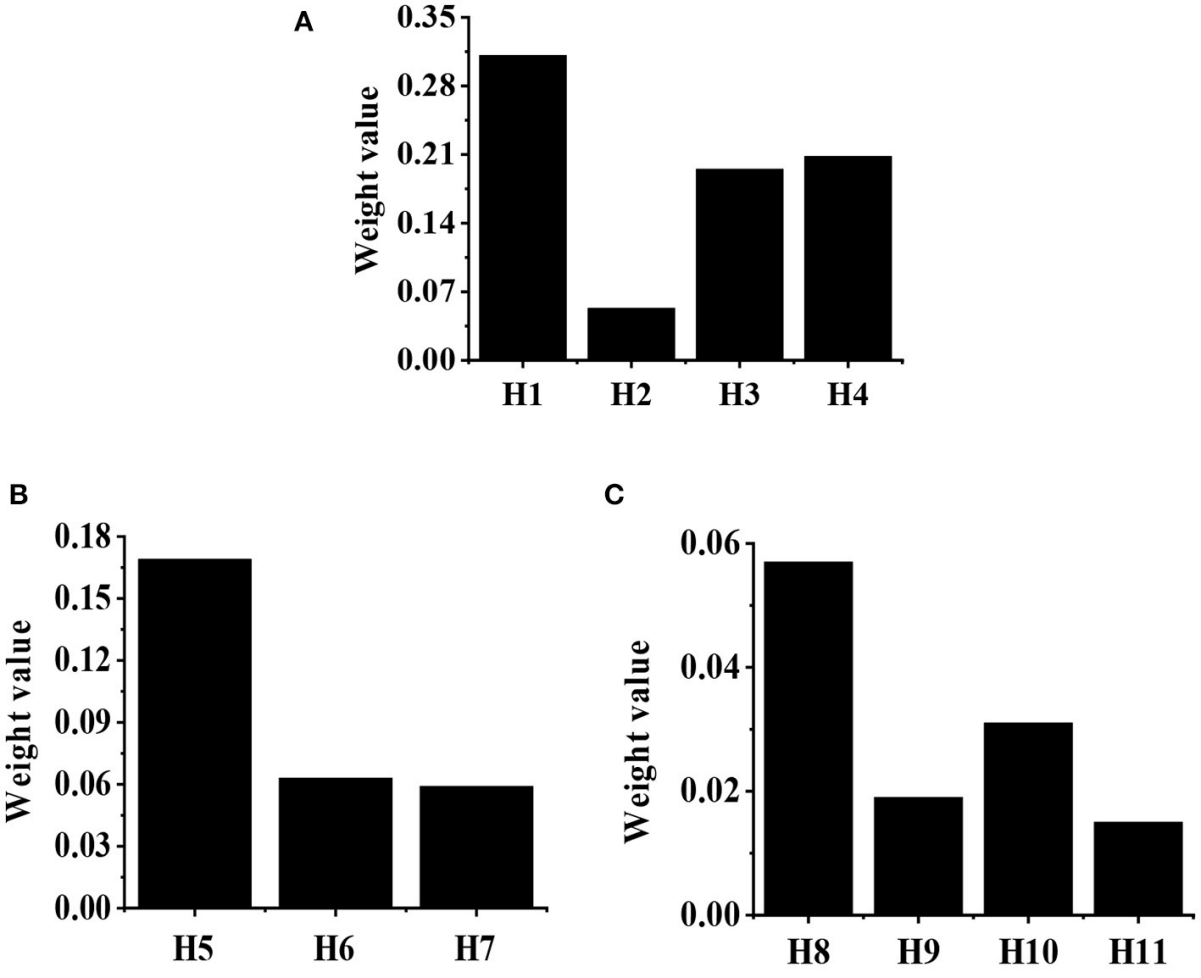
The weight value of each index in the index layer to the target layer W. **(A)** The index of G1; **(B)** the index of G2; **(C)** the G3 index.

According to weight values of the W–G and G–H judgment matrices obtained in 3.1.1 and 3.1.2, the weight value of each index in the index layer relative to the target layer W is calculated, as shown in Figure 10. In terms of G1, the weight value of H1 relative to target layer W is the largest (0.311), followed by the H4 index (0.208). In terms of G2, the weight value of the H5 index relative to target layer W is the largest (0.169). In terms of G3, the weight value of the H8 index relative to target layer W is the largest (0.057), followed by the H10 index (0.031).

### The Membership Degree of Each Index

#### The Membership Matrix of Each Index of G1

As shown in Table 6, the membership degree of H1 of G1 to the comment set is [0.481, 0.362, 0.157, 0], with 48.1% being excellent, 36.2% being good, and 15.7% being qualified. The membership degree of H2 to the comment set is [0.274, 0.405, 0.321, 0], with 27.4% being excellent, 40.5% being good, and 32.1% being qualified. The membership degree of H3 to the comment set is [0.288, 0.526, 0.186, 0], with 28.8% being excellent, 52.6% being good, and 18.6% being qualified. The membership degree of H4 to comment set is [0.433, 0.371, 0.196, 0], with 43.3% being excellent, 37.1% being good, and 19.6% being qualified.

**Table 6:** The membership matrix of each index of G1.

**Index**		**H1**	**H2**	**H3**	**H4**
Membership matrix	Excellent	0.481	0.274	0.288	0.433
	Good	0.362	0.405	0.526	0.371
	Qualified	0.157	0.321	0.186	0.196
	Unqualified	0	0	0	0

#### Membership Matrix of Each Index of G2

As shown in Table 7, the membership degree of H5 in G1 to the comment set is [0.247, 0.431, 0.240, 0.082], with 24.7% being excellent, 43.1% being good, 24% being qualified, and 8.2% being unqualified. The membership degree of H6 to comment set is [0.318, 0.377, 0.305, 0], with 31.8% being excellent, 37.7% being good, and 30.5% being qualified. The membership degree of H7 to comment set is [0.497, 0.311, 0.176, 0.016], with 49.7% being excellent, 31.1% being good, 17.6% being qualified, and 1.6% being unqualified.

**Table 7:** Membership matrix of each index of G2.

**Index**		**H5**	**H6**	**H7**
Membership matrix	Excellent	0.247	0.318	0.497
	Good	0.431	0.377	0.311
	Qualified	0.240	0.305	0.176
	Unqualified	0.082	0	0.016

#### Membership Matrix of Each Index of G3

As shown in Table 8, the membership degree of H8 of G3 to the comment set is [0.266, 0.608, 0.126, 0], with 26.6% being excellent, 60.8% being good, and 12.6% being qualified. The membership degree of H9 to the comment set is [0.198, 0.677, 0.128, 0], with 19.8% being excellent, 67.7% being good, and 12.8% being qualified. The membership degree of H10 to the comment set is [0.053, 0.811, 0.136, 0], with 5.3% being excellent, 81.1% being good, and 13.6% being qualified. Furthermore, the membership degree of H10 to the comment set is [0.038, 0.793, 0.169, 0], with 3.8% being excellent, 79.3% being good, and 16.9% being qualified.

**Table 8:** Membership matrix of each index of G3.

**Index**		**H8**	**H9**	**H10**	**H11**
Membership matrix	Excellent	0.266	0.195	0.053	0.038
	Good	0.608	0.677	0.811	0.793
	Qualified	0.126	0.128	0.136	0.169
	Unqualified	0	0	0	0

### Multilevel Fuzzy Comprehensive Evaluation Results

#### The Results of the First-Level Fuzzy Comprehensive Evaluation

As shown in Table 9, according to the first-level fuzzy equation, 41.20% of G1 is excellent, 35.80% is good, and 23% is qualified. For the G2, 39.5% is excellent, 34.7% is good, 15.1% is qualified, and 10.7% is unqualified. For G3, 22.7% is excellent, 40.5% is good, and 35.5% is qualified.

**Table 9:** Calculation of weight value of the G3–H judgment matrix.

**Index**		**G1**	**G2**	**G3**
Membership matrix	Excellent	0.412	0.395	0.227
	Good	0.358	0.347	0.405
	Qualified	0.230	0.151	0.355
	Unqualified	0	0.107	0

#### The Results of the Second-Level Fuzzy Comprehensive Evaluation

**Figure 11:**
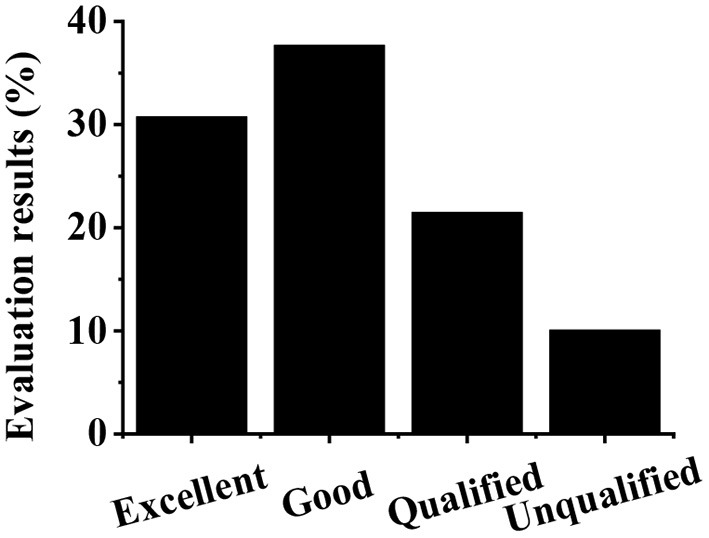
The results of the second-level fuzzy comprehensive evaluation.

As shown in Figure 11, according to the relative weight value of each index of the criterion layer to the target layer, the social benefit evaluation of the culture–tourism town is obtained through fuzzy calculation. Specifically, 30.76% of the social benefit of the culture–tourism town is excellent, 37.69% is good, 21.48% is qualified, and 10.07% is unqualified.

## Discussion

In recent years, the traditional sightseeing tourism, characterized by natural resources, is far from meeting people's growing demands on tourism. As the product of the tourism performing arts industry in the current culture–tourism integration, the culture–tourism town development is a new trend in tourism development (Law et al., 2019; Li et al., 2019). Based on deep learning BP neural network and fuzzy comprehensive analysis method, an artificial intelligence model for the culture–tourism town social benefit evaluation is constructed in the study. The weight values of G1, G2, and G3 relative to the target layer W are 0.522, 0.376, and 0.103, respectively, which is different from the research results of Saeidi et al. (2017). The development and construction of the culture–tourism town are found to have greatly improved the living standards of local residents, indicating that the weight value of life benefit is the largest, and it is an important factor in the evaluation of social benefit. The weight values of H1, H2, H3, and H4 relative to G1 are 0.507, 0.053, 0.296, and 0.144, respectively, indicating that the cultural inheritance and protection have the greatest influence on the evaluation results of G1 (Kulshrestha et al., 2020). The weight values of H5, H6, and H7 relative to G2 are 0.570, 0.092, and 0.338, respectively, which is consistent with the research results of Lv et al. (2018), indicating that the ecological environment improvement has the greatest influence on the evaluation results of G2. The weight values of H8, H9, H10, and H11 relative to the G3 are 0.424, 0.175, 0.218, and 0.186, respectively, indicating that the commercial economy development has the greatest influence on the evaluation results of G3 (Ma et al., 2018). According to the above analysis, the culture inheritance and protection, the ecological environment improvement, and the commercial economy development are the key factors to evaluate the social benefit of the culture–tourism town.

In addition, according to the first-level fuzzy comprehensive evaluation, for G1, 41.20% is excellent and 35.80% is good. For G2, 39.5% is excellent and 34.7% is good. For G3, 22.7% is excellent and 40.5% is good, which is different from the result obtained by Al Shehhi and Karathanasopoulos (2020). This may be due to the differences in the development priorities of towns with different culture–tourism characteristics. It is evident that G1 and G2 are excellent, while G3 is good. The culture inheritance and protection index has the highest weight value to the social benefit of the target layer, indicating that the construction of the culture–tourism town is conducive to carrying forward the traditional culture and the harmonious development of the society (Hossain and Muhammad, 2019; Wang, 2020). A fine ecological environment is a necessary condition for a culture–tourism town to satisfy tourists' viewing and behavioral psychological demands. G2 is found to be excellent for the subject town, indicating that the construction of a culture–tourism town causes little damage to the local environment (Farmaki, 2018). According to the second-level fuzzy evaluation results, 30.76% of the total social benefit of the culture–tourism town is excellent, 37.69% is good, 21.48% is qualified, and 10.07% is unqualified, which is similar to the research results of Cheng et al. (2017). The total social benefit is good, indicating that the development and construction of the culture–tourism town is of great significance.

## Conclusions

Based on deep learning BP neural network and fuzzy comprehensive analysis method, an artificial intelligence model is constructed for social benefit evaluation, with Yiyang town taken as the research subject. It is found that the life benefit and environmental benefit in the Yiyang culture–tourism town is excellent, with the economic benefit and the total social benefit being good. The culture inheritance and protection, the ecological environment improvement, and the commercial economy development are found to be the key factors to evaluate the social benefit of the culture–tourism town. However, some shortcomings are noted in this study. There are few previous references cited in this study, and the selection of social benefit factors of the culture–tourism town is subjective to some extent, which may reduce the power and increase the error margin of the results. More objective research is necessary in future. In conclusion, the study provides a theoretical basis for applying deep learning in social benefit evaluation of the culture–tourism integration industry.

## Data Availability Statement

The raw data supporting the conclusions of this article will be made available by the authors, without undue reservation.

## Ethics Statement

The studies involving human participants were reviewed and approved by Hunan City University Ethics Committee. The patients/participants provided their written informed consent to participate in this study. Written informed consent was obtained from the individual(s) for the publication of any potentially identifiable images or data included in this article.

## Author Contributions

The author confirms being the sole contributor of this work and has approved it for publication.

## Conflict of Interest

The author declares that the research was conducted in the absence of any commercial or financial relationships that could be construed as a potential conflict of interest.
